# Cardiorenal and other diabetes related outcomes with SGLT-2 inhibitors compared to GLP-1 receptor agonists in type 2 diabetes: nationwide observational study

**DOI:** 10.1186/s12933-021-01258-x

**Published:** 2021-03-22

**Authors:** Moa Lugner, Naveed Sattar, Mervete Miftaraj, Jan Ekelund, Stefan Franzén, Ann-Marie Svensson, Björn Eliasson

**Affiliations:** 1 Institute of Medicine, University of Gothenburg, Sahlgrenska University Hospital, Gothenburg, Sweden; 2 Institute of Cardiovascular and Medical Sciences, University of Glasgow, Glasgow, UK; 3 National Diabetes Register, Centre of Registers, Gothenburg, Sweden

**Keywords:** Type 2 diabetes, Epidemiology, Sodium glucose transporter 2 inhibitors, Glucagon‐like peptide-1 receptor agonist, Mortality, Cardiovascular disease

## Abstract

**Background:**

Major prospective randomized clinical safety trials have demonstrated beneficial effects of treatment with glucagon-like peptide-1 receptor agonists (GLP-1RA) and sodium–glucose co-transporter-2 inhibitors (SGLT-2i) in people with type 2 diabetes and elevated cardiovascular risk, and recent clinical treatment guidelines therefore promote early use of these classes of pharmacological agents. In this Swedish nationwide observational study, we compared cardiorenal outcomes and safety of new treatment with GLP-1RA and SGLT-2i in people with type 2 diabetes.

**Methods:**

We linked data from national Swedish databases to capture patient characteristics and outcomes and used propensity-score based matching to account for differences between the two groups. The treatments were compared using Cox regression models.

**Results:**

We identified 9648 participants starting GLP-1RA and 12,097 starting SGLT-2i with median follow-up times 1.7 and 1.1 years, respectively. The proportion of patients with a history of MACE were 15.8%, and 17.0% in patients treated with GLP-1RA and SGLT-2i, respectively. The mean age was 61 years with 7.6 years duration of diabetes. Mean HbA1c were 8.3% (67.6 mmol/mol) and 8.3% (67.2 mmol/mol), and mean BMI 33.3 and 32.5 kg/m^2^ in patients treated with GLP-1RA or SGLT-2i, respectively. The cumulative mortality risk was non-significantly lower in the group treated with SGLT-2i, HR 0.78 (95% CI 0.61–1.01), as were incident heart failure outcomes, but the risks of cardiovascular or renal outcomes did not differ. The risks of stroke and peripheral artery disease were higher in the SGLT-2i group relative to GLP-1RA, with HR 1.44 (95% CI 0.99–2.08) and 1.68 (95% CI 1.04–2.72), respectively.

**Conclusions:**

This observational study suggests that treatment with GLP-1RA and SGLT-2i result in very similar cardiorenal outcomes. In the short term, treatment with GLP-1RA seem to be associated with lower risks of stroke and peripheral artery disease, whereas SGLT-2i seem to be nominally associated with lower risk of heart failure and total mortality.

**Supplementary Information:**

The online version contains supplementary material available at 10.1186/s12933-021-01258-x.

## Background

Major prospective randomized clinical safety trials have demonstrated beneficial effects of treatment with glucagon-like peptide-1 receptor agonists (GLP-1RA) and sodium–glucose co-transporter-2 inhibitors (SGLT-2i) in people with type 2 diabetes and elevated cardiovascular risk [1, 2]. Recent clinical treatment guidelines therefore promote early use of these classes of pharmacological agents, particularly in patients with a history of cardiovascular disease (CVD), heart failure (HF), chronic kidney disease (CHD), or with multiple risk factors [3, 4]. These trials have all been based on placebo-controlled in design, included many participants, variable exposure time, and high statistical power. Epidemiological studies in wider populations in clinical practice have shown results supporting the above recommendations. Meta-analyses have generally supported cardiorenal benefits for SGLT-2i whereas GLP-1RA seem to lessen atherothrombotic outcomes more consistently [5, 6].

The 2018 American Diabetes Association (ADA) and European Association for the Study of Diabetes (EASD) consensus report stated that patients with type 2 diabetes and established atherosclerotic cardiovascular disease (ASCVD) should use GLP-1RA or SGLT-2i as part of glycaemic management [7]. Due to important research findings from recent large CVOT’s, the recommendations were updated 2019 [8]. The updated version of the consensus report declares that in high-risk individuals with type 2 diabetes, the decision to treat with a GLP-1RA or SGLT-2i should be considered independently of baseline HbA1c, as the cardiovascular benefits of these drugs are independent of HbA1c. The updated version also provides some guidance to which of the drug classes to choose depending on present risk factors. GLP-1RA is recommended in patients with type 2 diabetes and established ASCVD where major cardiovascular events (MACE) are considered the greatest threat, and in patients with indicators of high risk without established CVD, to reduce the risk of MACE. SGLT-2i on the other hand, are recommended in patients with type 2 diabetes and HF to reduce HF, MACE and CV death. SGLT-2i are also recommended in patients with type 2 diabetes and chronic kidney disease, to prevent progression of CKD, HF, MACE and CV death [8].

However, the CVOT’s the recommendations are based upon have recruited populations with somewhat differing cardiovascular diseases and cardiovascular risk factors, making it difficult to compare drug class effects. Head-to-head studies directly comparing the two drug classes regarding glycaemic control, body weight, blood pressure or cardiovascular outcomes are scarce. In the absence of such direct comparative trials, an observational study with inclusion of an unselected patient population with a great variation in clinical characteristics might help provide some more insight into potential differential effects of these two classes of drugs as they are prescribed in real life clinical practice. Such work could complement trial findings and aid clinical decision making when choosing between SGLT-2i and GLP-1RA.

The aim of this nationwide observational study based on The Swedish National Diabetes Register (NDR) was to examine outcomes and safety of treatment with GLP-1 receptor agonists compared with SGLT-2 inhibitors, in people with type 2 diabetes. We linked data from national Swedish databases to capture patient characteristics and outcomes and used propensity-score based matching to account for differences between the two groups. The treatments were compared using Cox regression models.

## Materials and methods

### Study design

This is a cohort study using nationwide register data from Sweden. We included patients with type 2 diabetes who were new users of any GLP-1RA, or any SGLT-2i, from July 9, 2013 through December 31, 2017. We defined the index date as the date of the first filled prescription of any drug of either of these classes of glucose-lowering medications. The clinical effects of the treatments as well as severe adverse events were captured using NDR, the national patient registry and the cause of death registry. The end of follow-up was 31 December 2017. All patients have consented to being reported in NDR, while no individual consent is required to be included in this study according to Swedish law. The regional ethical review board approved this study protocol.

### Data sources

The study database was created by use of the unique personal identity number assigned to every Swedish resident, as the identifier to cross-link national healthcare registries. The procedure as well as the anonymization of the data, was performed at the National Board of Health and Welfare. NDR is a national quality register, providing information reported by physicians and nurses on risk factors and complications of diabetes [9]. The NDR is estimated to include almost 90% of all patients with type 2 diabetes aged ≥ 18 years in Sweden. We used data on clinical characteristics from NDR. Information on pre-existing comorbidities (hospitalizations from 1997 to index date), and conditions occurring during the study period, including severe hypoglycaemia and hyperglycaemia and carry data such as date of contact, diagnostic codes, and procedure codes, were obtained from the national patient register. The prescribed drug register contains information on all prescriptions, including information on specific drug, date of drug dispensing, and amount of drug that have been filled at a pharmacy after 1/7/2005. All medications are classified according to the Anatomical Therapeutic Chemical (ATC) classification system and the cause of death registry holds information on mortality (date and diagnosis). Data on socioeconomic variables (education level, marital status, available income, born in or outside Sweden) was retrieved from the Longitudinal Integration Database for Health Insurance and Labor market Studies. These national databases have complete and nationwide coverage and have previously been described validated [10].

### Participants

We used the following criteria for inclusion in the study: male and female patients 18 years of age or older, with type 2 diabetes as determined clinically by the reporting centers. The upper BMI limit was 45 kg/m^2^, and the estimated glomerular filtration rate (eGFR, according to Modification of Diet in Renal Disease, MDRD, formula) was 30 ml/min/1.73 m^2^) or higher. We excluded from the analysis people with a history of pancreatitis, cancer during 5 years preceding the index date, renal or liver transplantation, dialysis or bariatric surgery, as well as all people with any filled prescription of any GLP-1RA (ATC codes A10BJ01-05), SGLT-2i (ATC codes A10BK01-4), or insulin (ATC codes A10A), from 1/7/2005 through the index date.

### Outcomes

We monitored the participants from the index date until the first incident of hospitalizations due to any outcome, death, or until Dec 31, 2017. The outcomes were total mortality, MACE, fatal or non-fatal CVD including myocardial infarction and stroke, HF, severe renal disease and transplantation, hyper- or hypoglycaemia, ketoacidosis, diabetic nephropathy or retinopathy. A table of outcome definitions is provided in Additional file 1.

### Statistical analysis

The primary analysis was done in accordance with the intention-to-treat principle. Thus, participants were classified as exposed or non-exposed at the index date and this classification was then used throughout follow-up. We also examined the effects in people with continuous use of the new treatment, defined as at least two filled prescriptions per year, until censoring (change to, or addition of, another class of glucose-lowering treatment) or to the end of follow-up.

To compare the effects in the two treatment groups, we used a propensity score adjusted analysis using inverse probability of treatment weighting (IPTW) to estimate the average treatment effect for everyone (ATE). The propensity scores were estimated using a generalized boosted binomial regression model with an interaction depth of 3, a maximum of 10,000 trees, and a shrinkage of 0.01. The optimal number of trees was selected using a stopping rule applied to the degree of balance. The full list of variables included in the generalized boosted binomial regression model is provided in Additional file 2. The propensity scores were estimated separately for 10 imputed data sets and the average probability was then converted to an analysis weight estimating the ATE. The weighted descriptive statistics are presented with standardized mean differences.

Number of events, person years, and incidence rate per 1000 patient-years are given with exact 95% Poisson confidence interval. The time to event is described graphically using an unweighted Kaplan–Meier estimator. The treatments were compared using crude and IPTW Cox regression with treatment group as the only independent variable.

Results were considered statistically significant if the 95% confidence intervals (CI) did not overlap 1.0. We used SAS version 9.4 and R version 3.5.1.

## Results

We identified 9648 participants starting treatment with a GLP-1RA and 12,097 starting treatment with a SGLT-2i. The median follow-up time were 1.7 and 1.1 years, respectively. The three most used GLP-1RA were liraglutide (75.1%, dulaglutide (16.3%), and exenatide once weekly (6.4%). Empagliflozin was used by 56.6% in SGLT-2i group, while 43.2% used dapagliflozin and 0.2% used canagliflozin.

The baseline characteristics of the participants are given in Table 1. The propensity score-adjusted analysis using IPTW showed none or minimal differences in clinical characteristics, including history of previous conditions such as cardiovascular disease or socioeconomic variables, between the groups (all standardized mean differences > 0.2). The mean age was 61 years with 7.6 years mean duration of diabetes and 38% of the patients were women. The mean HbA1c were 8.3% (67.6 mmol/mol) and 8.3% (67.2 mmol/mol) in patients treated with GLP-1RA or SGLT-2i, respectively, and the mean BMI were 33.4 kg/m^2^ and 32.4 kg/m^2^, respectively. The mean levels of blood pressure and LDL cholesterol were 135/80 mmHg and 2.6 mmol/L with no differences between the groups, and the mean eGFR levels were 91.3 and 92.1 (ml/min/1.73 m^2^), respectively. Micro-, macroalbuminuria and smoking did not differ meaningfully either. The proportion of patients with a history of MACE were 15.8%, and 17.0% in patients treated with GLP-1RA and SGLT-2i, respectively, and there were also only modest differences in the use of metformin, other glucose-lowering, antihypertensive or lipid-lowering medications between the groups (Table 1).

**Table 1:** Clinical characteristics of participants starting treatment with a GLP-1RA or a SGLT-2i

Variable	GLP-1RA (n = 9684)	SGLT-2i (n = 12,097)	P value	Standardized mean difference
Sex (female %)	38.1	37.0	0.152	0.022
Age (years)	60.23 (11.00)	60.82 (10.92)	0.001	0.054
Diabetes duration (years)	7.42 (5.67)	7.67 (5.79)	0.012	0.044
HbA1c (mmol/mol)	67.55 (14.75)	67.20 (14.49)	0.175	0.024
BMI (kg/m^2^)	33.40 (6.04)	32.41 (5.90)	< 0.001	0.166
Systolic blood pressure (mmHg)	135.17 (14.70)	135.22 (15.07)	0.856	0.003
Diastolic blood pressure (mmHg)	80.46 (9.63)	80.21 (9.77)	0.122	0.026
LDL cholesterol (mmol/L)	2.56 (0.95)	2.55 (0.95)	0.461	0.014
HDL cholesterol (mmol/L)	1.13 (0.31)	1.14 (0.32)	0.009	0.051
Triglycerides (mmol/L)	2.32 (1.58)	2.29 (1.66)	0.217	0.024
eGFR (ml/min/1.73 m^2^)	91.32 (24.58)	92.05 (24.07)	0.087	0.030
Microalbuminuria (%)	20.7	20.6	0.869	0.003
Macroalbuminuria (%)	4.5	4.1	0.250	0.023
Smoker (%)	15.1	15.5	0.551	0.011
History of MACE (%)	15.8	17.0	0.043	0.032
Heart failure (%)	3.5	3.8	0.353	0.014
Metformin treatment (%)	87.3	86.6	0.175	0.021
Sulfonylurea treatment (%)	29.3	29.0	0.610	0.008
Alpha-glucosidase inhibitor treatment(%)	0.4	0.4	0.412	0.012
Meglitinide treatment (%)	5.2	5.1	0.859	0.003
Thiazolidinedione treatment (%)	1.6	1.7	0.967	0.001
Antihypertensive medication (%)	73.9	73.9	0.986	0.001
RAS blocker (%)	65.7	65.7	0.979	< 0.001
Lipid-lowering medication (%)	65.0	65.3	0.705	0.006

Propensity score adjusted analysis using inverse probability of treatment weighting estimating the average treatment effect for everyone. Means with standard deviations. Numbers and proportions (%)

*MACE* major adverse cardiovascular events

**Fig. 1:**
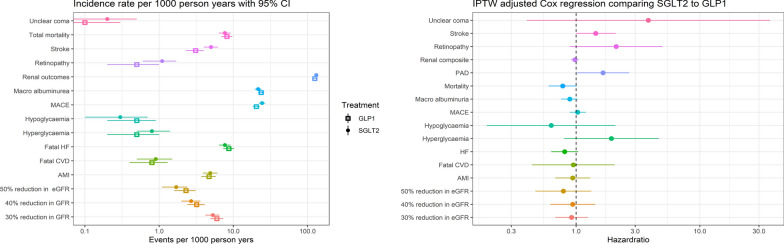
Outcome analysis. Results of Cox analysis on primary and secondary outcomes. Event rates are reported as number of events/1000 person-years

Table 2 and Fig. 1 gives numbers of events, incidence rates, and hazard ratios (HR) for the outcomes in patients treated with SGLT-2i vs. GLP-1RA based on IPTW-adjusted Cox regressions. The incidence rates of total mortality, fatal HF, severe hypoglycemia, as well as halved eGFR and macroalbuminuria, were non-significantly higher in the group treated with GLP-1RA than in the group treated with SGLT-2i. The incidence rates of stroke and retinopathy were lower in patients treated with a GLP-1RA than in patients treated with a SGLT-2i. The incidence rates of ketoacidosis were low and similar in the two groups. However, there were no marked differences in incidence rates of any outcomes between patients treated with either a SGLT-2i or a GLP-1RA (all 95% confidence intervals overlapping) (Table 2).

**Table 2:** Number of events and event rates in participants after starting treatment with a GLP-1RA or a SGLT-2i and IPTW adjusted Cox regressions comparing SGLT-2i to GLP-1RA

Outcome	GLP-1RA		SGLT-2i		SGLT-2i vs. GLP-1RA	
	Events	Incidence rate	Events	Incidence rate	Hazard ratio, c	P-value
Total mortality	145	8.2 (6.9–9.7)	128	7.7 (6.4–9.2)	0.78 (0.61–1.01)	0.0641
MACE	348	20.4 (18.3–22.7)	392	24.4 (22.0–26.9)	1.03 (0.89–1.21)	0.6608
Fatal CVD	14	0.8 (0.4–1.3)	15	0.9 (0.5–1.5)	1.00 (0.47–2.13)	0.9947
Myocardial infarction	82	4.7 (3.7–5.8)	81	4.9 (3.9–6.1)	0.94 (0.68–1.3)	0.7295
Stroke	54	3.1 (2.3-4.0)	82	5.0 (4.0–6.2)	1.44 (0.99–2.08)	0.0562
Fatal CHF	151	8.7 (7.3–10.2)	127	7.7 (6.4–9.2)	0.83 (0.65–1.07)	0.1503
Renal composite	1864	125.2 (119.6–131.0)	1882	131.1 (125.3–137.2)	0.98 (0.92–1.05)	0.5676
30% reduction eGFR	105	6 (4.9–7.3)	87	5.3 (4.2–6.5)	0.92 (0.68–1.25)	0.5863
40% reduction eGFR	56	3.2 (2.4–4.1)	45	2.7 (2.0–3.6)	0.94 (0.62–1.43)	0.7718
Halved eGFR	40	2.3 (1.6–3.1)	28	1.7 (1.1–2.4)	0.80 (0.47–1.35)	0.4007
Macroalbuminuria	403	23.7 (21.5–26.1)	349	(21.6 (19.4–24.0)	0.89 (0.77–1.04)	0.1408
Retinopathy	9	0.5 (0.2–1.0)	18	1.1 (0.6–1.7)	2.15 (0.92–5.03	0.0784
Hypoglycemia	8	0.5 (0.2–0.9)	5	0.3 (0.1–0.7)	0.69 (0.21–2.28)	0.5392
Hyperglycemia	9	0.5 (0.2-1.0)	14	0.8 (0.5–1.4)	1.88 (0.78–4.52)	0.1571
Ketoacidosis	1	0.1 (0.0-0.3)	1	0.1 (0.0-0.3)	1.68 (1.04–2.72)	0.0346

Incidence rate per 1000 patient-years with exact 95% Poisson confidence intervals. MACE, major adverse cardiovascular events. CVD, cardiovascular disease. CHF, congestive heart failure. Renal composite, any of micro- or macroalbuminuria, eGFR 50% reduction or lower than 60, dialysis, renal transplantation, renal failure, renal death. The IPTW statistical analysis is based on Cox regressions with exposure as the only covariate. The weights are defined to estimate the average treatment effect for everyone (ATE)

**Fig. 2:**
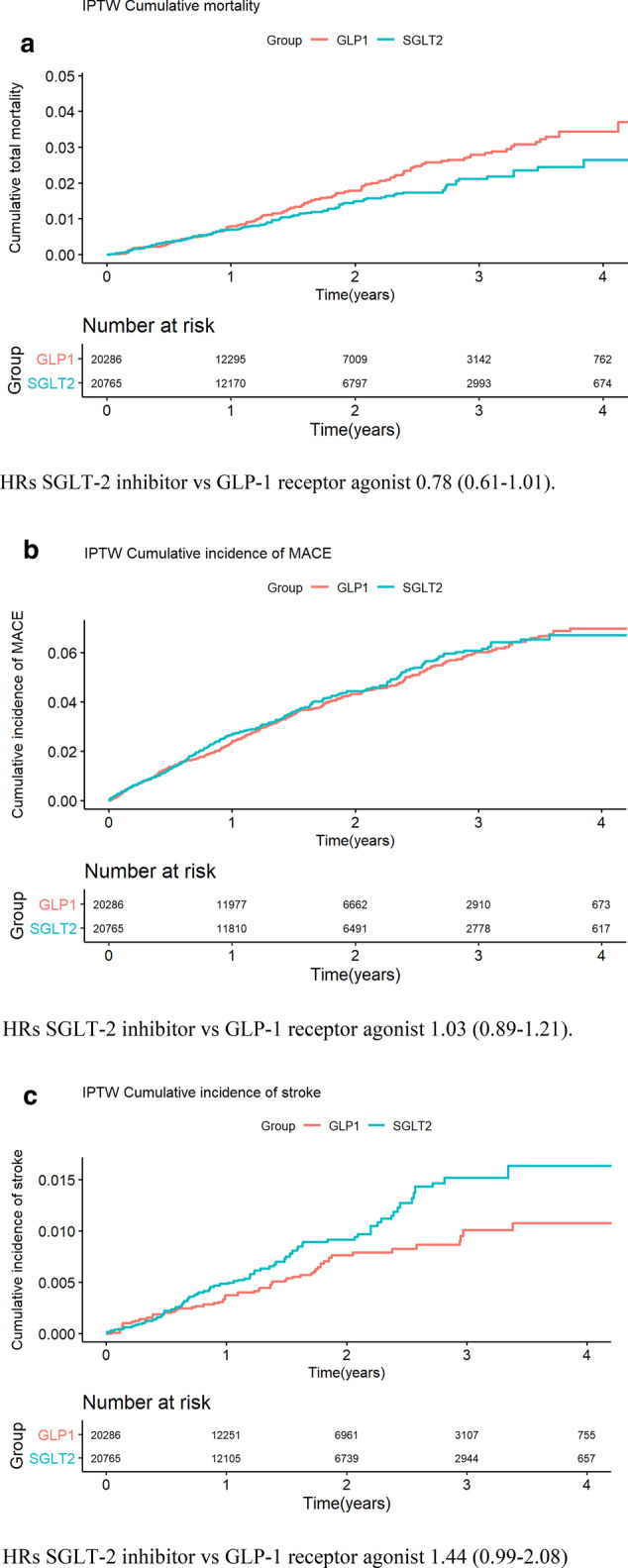

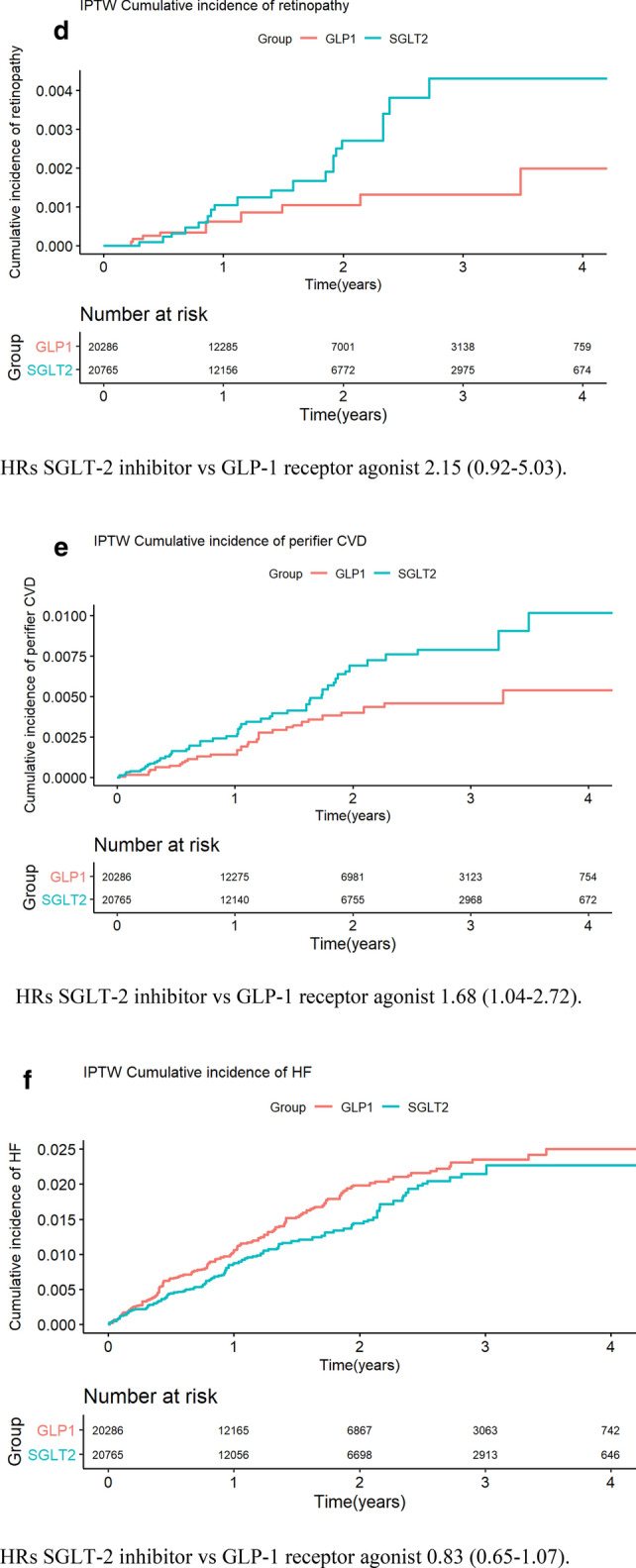
**a** Cumulative total mortality (IPTW) after starting treatment with a GLP-1RA or a SGLT-2i. **b** Cumulative incidence rates of MACE (IPTW) after starting treatment with a GLP-1RA or a SGLT-2i. HRs SGLT-2 inhibitor vs. GLP-1 receptor agonist 1.03 (0.89–1.21). ** c** Cumulative incidence stroke (IPTW) after starting treatment with a GLP-1RA or a SGLT-2i. HRs SGLT-2 inhibitor vs. GLP-1 receptor agonist 1.44 (0.99–2.08). **d** Cumulative incidence rates of retinopathy (IPTW) after starting treatment with a GLP-1RA or a SGLT-2i. HRs SGLT-2 inhibitor vs. GLP-1 receptor agonist 2.15 (0.92–5.03). **e** Cumulative incidence rates of peripheral artery disease (IPTW) after starting treatment with a GLP-1RA or a SGLT-2i. HRs SGLT-2 inhibitor vs. GLP-1 receptor agonist 1.68 (1.04–2.72). **f** Cumulative incidence rates of congestive heart failure (IPTW) after starting treatment with a GLP-1RA or a SGLT-2i. HRs SGLT-2 inhibitor vs. GLP-1 receptor agonist 0.83 (0.65–1.07)

Figure 2a–f show Kaplan–Meier graphs illustrating cumulative total mortality and incidence rates of MACE, stroke, retinopathy, peripheral CVD and HF in the two treatment groups. The cumulative mortality risk was non-significantly lower in the group treated with SGLT-2i, HR 0.78 (95% CI 0.61–1.01). The risks of MACE, acute myocardial infarction, CVD, and renal composite were similar between the two groups with HRs close to 1. The risks of HF and severe hypoglycemia were non-significantly lower in the group treated with SGLT-2i, HR 0.83 (95% CI 0.65–1.07) and 0.69 (95% CI 0.21–2.28), respectively. The risks of stroke and peripheral artery disease (PAD) were higher in the group treated with SGLT2-i relative to the group treated with GLP1-RA, HR 1.44 (95% CI 0.99–2.08) and 1.68 (95% CI 1.04–2.72), respectively. HR is however only nominally significant in PAD (p-value = 0.035).

The reduction of HbA1c and systolic blood pressure were similar between the two groups. In the group treated with GLP-1RA the reduction in HbA1c was 10.05 mmol/mol (95% CI 10.68–9.43) 12 months post index date, while the corresponding reduction for the group treated with SGLT-2i was 9.15 mmol/mol (95% CI 9.80–8.49). The group treated with SGLT-2i had a mean weight loss of 3.46 kg (95% CI 3.77–3.14), while those treated with GLP-1RA lost in average 2.49 kg (95% CI 2.76–2.22).

Subgroup analyses were performed with IPTW adjusted cox-regressions. The stratification was made according to pre-existing CVD, pre-existing HF, pre-existing cardiorenal disease (i.e., a composite of eGFR < 60, micro- or macroalbuminuria, MACE, AMI, stroke, or CVD), and albuminuria (Table 3). Patients treated with GLP-1RA had significantly lower risk of stroke in the group without established CVD compared to SGLT-2i, HR 1.73 (95% CI 1.11–2.68), as well as in the group without pre-existing cardiorenal disease, HR 2.08 (95% CI 1.21–3.58). The risk of MACE in those without established CVD was also lower for GLP-1RA, HR 1.27 (95% CI 1–1.62), however underpowered (p = 0.053) (Table 3).

**Table 3:** IPTW adjusted Cox regressions comparing SGLT-2i to GLP-1RA after stratification according to pre-existing CVD, pre-existing cardiorenal disease, pre-existing HF, and pre-existing albuminuria

Outcome		Total mortality	MACE	Fatal CVD	Myocardial infarction	Stroke	HF	Renal composite	PAD
Pre-index CVD	HR (95% CI)	0.65 (0.40–1.05)	0.9 (0.74–1.10)	1.95 (0.47–8.12)	0.94 (0.57–1.54)	1.03 (0.54–1.98)	0.79 (0.54–1.16)	1.05 (0.89–1.22)	2.56 (1.09–6.01)
	P-value	0.080	0.303	0.356	0.792	0.927	0.238	0.584	0.030
Free from pre-index CVD	HR (95% CI)	0.86 (0.64–1.15)	1.27 (1.00-1.62)	0.69 (0.27–1.77)	0.94 (0.62–1.43)	1.74 (1.11–2.68)	0.84 (0.60–1.17)	0.97 (0.90–1.04)	1.37 (0.77–2.46)
	P-value	0.300	0.053	0.447	0.768	0.014	0.295	0.399	0.288
Pre-index cardiorenal disease	HR (95% CI)	0.71 (0.49–1.02)	0.93 (0.77–1.12)	1.28 (0.44–3.70)	1.01 (0.63–1.63)	1.07 (0.65–1.75)	0.8 (0.57–1.11)	0.93 (0.85–1.01)	2.03 (1.05–3.91)
	P-value	0.067	0.422	0.648	0.968	0.792	0.176	0.090	0.034
Free from pre-index cardiorenal disease	HR (95% CI)	0.91 (0.65–1.29)	1.30 (0.99–1.71)	0.68 (0.22–2.14)	0.88 (0.57–1.37)	2.08 (1.21–3.58)	0.81 (0.55–1.19)	1.01 (0.91–1.12)	1.44 (0.71–2.91)
	P-value	0.604	0.056	0.513	0.577	0.008	0.281	0.828	0.316
Pre-index HF	HR (95 % CI)	0.80 (0.46–1.39)	0.99 (0.65–1.49)	1.88 (0.36–9.87)	0.86 (0.29–2.56)	1.74 (0.40–7.45)	0.92 (0.64–1.31)	0.97 (0.73–1.29)	16.47 (1.99-136.06)
	P-value	0.426	0.954	0.458	0.793	0.459	0.636	0.821	0.009
Free from pre-index HF	HR (95% CI)	0.81 (0.61–1.07)	1.04 (0.89–1.23)	0.91 (0.39–2.10)	0.95 (0.68–1.33)	1.42 (0.97–2.08)	0.79 (0.56–1.11)	0.98 (0.92–1.05)	0.79 (0.56–1.11)
	P-value	0.138	0.610	0.820	0.744	0.072	0.173	0.625	0.173
Pre-index albuminuria	HR (95 % CI)	0.76 (0.43–1.35)	1.01 (0.69–1.49)	1.25 (0.29–5.28)	1.18 (0.44–3.22)	1 (0.47–214)	1.38 (0.79–2.42)	0.91 (0.82-1)	2.08 (0.75–5.74)
	P-value	0.3506	0.9418	0.7636	0.7420	0.9980	0.2630	0.0539	0.1577
Free from pre-index albuminuria	HR (95% CI)	1.01 (0.65–1.57	1.24 (0.97–1.6)	0.91 (0.2–4.07)	0.9 (0.57–1.48)	2.02 (1.09–3.76)	0.91 (0.58–1.43)	1.21 (1.05–1.4)	2.44 (1.11–5.38)
	P-value	0.9716	0.0912	0.8996	0.6672	0.0261	0.6788	0.0077	0.0265

Cardiorenal composite, any of eGFR < 60, micro- or macroalbuminuria and any MACE, AMI, stroke, or CVD. Renal composite, any of micro- or macroalbuminuria, eGFR 50% reduction or eGFR < 60, dialysis, renal transplantation, renal failure, renal death

HRs SGLT-2 inhibitor vs. GLP-1 receptor agonist 0.78 (0.61–1.01)

In continuous users, the results were overall in broad agreement with those seen in the ITT population. The cumulative mortality rate was significantly lower for SGLT-2i compared to GLP-1RA with HR 0.72 (95% CI 0.55–0.94). HR for MACE was 1.0 (95% CI 0.84–1.18), and HR for AMI was 0.87 (95% CI 0.61–1.24). HR for stroke was 1.52 (95% CI 0.97–2.39) and HR for PAD was 1.77 (95% CI 1.03–3.06). HR for HF was 0.82 (95% CI 0.61–1.08) and HR for renal composite was 0.96 (95% CI 0.89–1.03).

## Discussion

GLP-1RA and SGLT-2i are being increasingly used in people with type 2 diabetes, due to efficacy and safety data in extensive clinical trial programs. This is also in line with current clinical treatment recommendations (ADA/EASD, ESC) [3, 4]. Recent major cardiovascular outcome trials have shown quite comparable effects for these two classes of drugs in patients with type 2 diabetes with established cardiovascular disease or multiple risk factors, although hinting at better cardiorenal outcomes in the SGLT-2i class and more consistent atherothrombosis for the GLP-1RA class [1, 2, 11]. The present observational study including almost 22,000 patients with type 2 diabetes, show similar outcome rates of MACE, AMI and fatal CVD when initiating therapy with either SGLT-2i or GLP-1RA. However, it did strongly hint at better potential and quite rapid effects of GLP-1RA on stroke and PAD, whereas total mortality and HF point estimates were lower for the SGLT-2i class, as they were for some renal outcomes.

These results are consistent with previous research, showing that the two drug classes reduce the risk of MACE to a comparable extent [5, 12]. Both have also been shown to significantly reduce the risks of AMI and fatal CVD [5]. Although these results suggest that SGLT-2i and GLP-1RA have quite similar effects, we also found some numerically but statistically non-significant trends where they differentiated. The risk of heart failure was lower with SGLT-2i in our study. SGLT-2i’s effectiveness preventing heart failure has been demonstrated previously [13–16]. Recent evidence also shows benefits on progression of HF when treating patients with existing HF at baseline [17, 18]. By contrast, a meta-analysis suggested only a borderline benefit on HF prevention in the GLP-1RA class [19]. The risks of PAD and stroke on the other hand where lower with GLP-1RA in the present study. Furthermore, the overall mortality risk seemed to be lower when treating with a SGLT-2i than with a GLP-1RA. Whilst this is notable, it may be the short-term follow-up meant that the GLP-1RA class did not have a chance to lower total mortality, which was seen in the meta-analysis of available trial evidence [1]. In the subgroup analyses, GLP-1RA’s positive effect on stroke was especially pronounced for those without pre-existing CVD or pre-existing cardiorenal disease.

In the present study, the effects on metabolic endpoints such as HbA1c, blood pressure and body weight were comparable between the two treatment groups. Both groups lost weight, approximately 2.5 kg for the group treated with GLP-1RA and around 3.5 kg for the group treated with SGLT-2i. These results are comparable to the weight loss achieved in available Phase III trials [20–22]. The group treated with GLP-1RA had a slight greater reduction in Hba1c. Although the differences were small between the groups in the present study, a recent meta-analysis has shown that GLP-1RA might be somewhat more effective reducing HbA1c than SGLT-2i [23].

Even though the results are similar for many of the outcome measures, SGLT-2i and GLP-1RA have completely different mechanisms of actions. SGLT-2i lowers plasma glucose, weight, and blood pressure through their glucosuric and diuretic effects [24]. However, modulation of renal hemodynamics, preservation of renal function, improvement of salt and water homeostasis, as well as reduced sympathetic activation and inflammation have all been suggested to contribute [24]. Activation of the GLP-1 receptor also leads to multiple beneficial, direct or indirect, cordial and endothelial effects. These include reduced inflammation, less ischemic injury, improved endothelial function, vasodilatation and blood flow, but less smooth muscle proliferation and platelet aggregation. Collectively, these effects may have better benefits on atherothrombotic outcomes [25]. These are to be added to the well-known metabolic effects on glucagon as well as glucose-dependent insulin production, appetite regulation and gastric emptying, with positive consequences on glycemic and weight control [26]. Both SGLT-2i and GLP-1RA have been suggested to improve ventricular remodeling in patients with type 2 diabetes, an important determinant of morbidity and long-term outcome [27], with robust trial evidence for SGLT2i recently emerging in patients with existing heart failure and reduced ejection fraction, with reductions in ventricular volumes [28]. Since these drug classes has different mechanisms of action, there is a growing interest of the use of them in conjunction with each other to achieve synergistic effects. Though this area needs to be examined in more detail, results suggest that co-treatment with SGLT-2i and GLP-1RA might attain beneficial effects on cardiovascular endpoints as well as on glycemic control and seems to be well tolerated by patients [29, 30].

In the present study, we chose a new-user design to avoid selection and immortal time biases. This approach has been advocated recently [31], and all confounders were assessed prior to exposure. This was determined prior to the start of the follow-up, thus minimizing the risk of reverse causality. The risk of bias due to confounding is mitigated using IPTW. Compared to matching this makes it easier to control the estimand, in this case the ATE. An alternative would have been to use the common “greedy” 1–1 matching on propensity scores, which would have estimated the average effect for the treated (ATT). However, in that case we would have needed to define one of GLP-1RA or SGLT-2i as the “treated group” and the other as the “control group”, making this method non-optimal for this kind of pairwise comparison. The risk of IPTW is that you can get very large weights for some people, resulting in inflated standard errors. We performed an IPTW adjusted Cox regressions using truncated IPTW (ATE) weights, with minimal changes from the non-truncated analyse. This indicates that the weights were well behaved in the comparison between the GLP-1RA and SGLT-2i (Fig. 2).

### **Study limitations**

As always with observational studies, there is a possibility of confounding by indication making the treatment groups differ in some instances at baseline. We have addressed this problem with IPTW, a well-known strategy to obtain a pseudo-randomization in observational studies, ensuring the best possible balance between the groups. Another limitation of this study is the follow-up time. Results from CVOT’s imply that SGLT-2i exert its positive effects more rapidly than GLP-1RA, why a longer follow-up time might have influenced the results in some instances [14, 32]. Some of our results were underpowered and did not reach the conventional level of significance (p = 0.05) but were acknowledged and discussed anyhow since there was high pre-test probability from trial results supporting them and the noticed difference in effect between the drug classes were large. As discussed in the article by Sterne and Smith, the p value needs to be interpreted in the given context and should not be used alone as a dichotomous tool to decide whether to discard a result as meaningful or not [33].

A major strength of the present study is its nationwide scope, based on registries and databases with almost complete national coverage. Another advantage is the inclusion of an unselected patient population with a great variation in clinical characteristics, and only few exclusion criteria, i.e., BMI higher than 45 kg/m^2^, eGFR lower than 30 ml/min/1.73 m^2^, a history of pancreatitis, recent cancer, renal or liver transplantation, dialysis or bariatric surgery.

## Conclusions

The results of this observational pharmacoepidemiological study suggests that GLP-1RA and SGLT-2i are very similar with respect to risks of several major cardiovascular outcomes, such as MACE, acute myocardial infarction, and fatal CVD. Treatment with GLP-1RA seems to be associated with lower risks of PAD and stroke (including in those without prior ASCVD), whereas there is some hint of lower HF and total mortality in the short term in those prescribed SGLT-2i’s. In the absence of dedicated head-to-head clinic trials, these results might provide further guidance for clinicians choosing between the two classes of drugs in patients with type 2 diabetes.

## Supplementary Information

**Additional file 1.** Table with definitions of the different outcome categories used in the analyses.

**Additional file 2.** Full list of variables included in the propensity score model.

## Data Availability

Not applicable.

## Abbreviations

GLP-1RA: Glucagon-like peptide-1 receptor agonists
SGLT-2i: Sodium–glucose co-transporter-2 inhibitors
ASCVD: Atherosclerotic cardiovascular disease
MACE: Major cardiovascular events
HF: Heart failure
CKD: Chronic kidney disease
NDR: National Diabetes Register of Sweden
BMI: Body mass index
