# Supplementary material for: Cardiorenal and other diabetes related outcomes with SGLT-2 inhibitors compared to GLP-1 receptor agonists in type 2 diabetes: nationwide observational study
Source: Cardiovasc Diabetol. 2021 Mar 22;20:67. doi: 10.1186/s12933-021-01258-x (PMC7983265; doi:10.1186/s12933-021-01258-x)
Supplement: Supplementary file 1 — Additional file 1. Table with definitions of the different outcome categories used in the analyses. [file 12933_2021_1258_MOESM1_ESM.docx]

| Outcome | ICD10 | Definition |
| --- | --- | --- |
| Acute myocardial infarction (AMI) | I21... |  |
| Coronary artery disease (CAD) | I22..., I23..., I24..., I25... |  |
| Stroke | I61..., I62..., I...,63 |  |
| Fatal CVD (cause of death) | I21..., I61..., I62..., I63..., I64... |  |
| Macroalbuminuria |  | at least two lab test within one year with albumin/creatinine ratio > 30 mg/mmol (or U-albumin >200µg/min, or > 300 mg/l) |
| Microalbuminuria |  | at least two lab test within one year with albumin/creatinine ratio 3-30  mg/mmol (or U-albumin 20-200µg/min, or  20-300 mg/l) |
| Halved eGFR |  | 50% reduction from baseline |
| Dialysis | Z490, Z491, Z492, Z992, DR015, DR016, DR023, DR024 |  |
| Renal failure | N17…, N18…, N19…, N99…, E112, E102, N083, Z940, KAS00, KAS10, KAS20 |  |
| Renal death (cause of death) | N17…, N18…, N19…, N99…, E112, E102, N083, Z940, KAS00, KAS10, KAS20 |  |
| Renal transplantation | Z940, KAS00, KAS10, KAS20 |  |
| Renal composite |  | micro- or macroalbuminuria, 50% reduction in eGFR or eGFR < 60, dialysis, renal transplantation, renal failure, renal death |
| MACE | I21…, I20…, I22…, I23…, I24…, I25…, I61…, I62…, I63…, I64…, |  |
| Fatal MACE (cause of death) |  | MACE or fatal CVD |
| Retinopathy | E103A, E103B, E103C, E113A, E113B, E113C, H360, H360A, H360B, H360X |  |
| Hypoglycaemia | E160, E161, E161W, E162, E100C, E110C, E106A, E116A |  |
| Hyperglycemia | E100A, 'E100B, E100D, E101, E101A, E101B, E101D, E101X, E110A, E110B, E110D, E111, E111A, E111B, E111D, E111X, E121, E131, E141, R739 |  |
| Ketoacidosis | E100A, E100B, E100C, E100D, E100X, E101A, E101B, E101, E101D, E101X |  |
