# Supplementary material for: Cardiorenal and other diabetes related outcomes with SGLT-2 inhibitors compared to GLP-1 receptor agonists in type 2 diabetes: nationwide observational study
Source: Cardiovasc Diabetol. 2021 Mar 22;20:67. doi: 10.1186/s12933-021-01258-x (PMC7983265; doi:10.1186/s12933-021-01258-x)
Supplement: Supplementary file 2 — Additional file 2. Full list of variables included in the propensity score model. [file 12933_2021_1258_MOESM2_ESM.docx]

**List of variables included in the propensity score model:**

Acute myocardial infarction

Alpha-glucosidase inhibitors

Amputation

Antihypertensive drugs

Atrial fibrillation

BMI

Born in sweden

Coronary artery disease

Diastolic blood pressure

Dipeptidyl Peptidase-4

Disposable income

Duration of diabetes

Educational level

eGFR

Gastrointestinal disorders

HbA1c

HDL

Heart failure

Hyperglycemia

Hyperlipidemia

Hypertension

Hypoglycemia

Index age

Index year

Ketoacidosis

LDL

Lipid lowering agent

Lipids

MACE

Macroalbuminuria

Manifest Cardiovascular disease

Marital status

Meglitinides

Mental disorder

Metformin

Microalbuminuria

Nephropathy

Neuropathy

Obesitas

Other glucose-lowering agents

Others (ICD10-codes: I200, I240, I241, I248, I249, I252)

Peripheral artery disease

Physical activity

Retinopathy

Sex

Smoker

Stroke

Sulfonylurea

Systolic blood pressure

Thiazolidinedione

Triglycerides

Unclear coma

Unstable angina pectoralis
